# Experience With Radical Resection in The Management of Proximal Bile Duct Cancer

**DOI:** 10.1155/1989/37642

**Published:** 1989

**Authors:** Jean C. Emond, James T. Mayes, Dale A. Rouch, J. Richard Thistlethwaite, Christoph E. Broelsch

**Affiliations:** University of Chicago Pritzker School of Medicine Department of Surgery Chicago IL 60637 USA

## Abstract

Multiple surgical and nonsurgical approaches have been advocated for the treatment of proximal bile duct cancer. However, survival appears longest when a resection can be performed. Fifteen patients treated at a university center were managed with an aggressive surgical approach. Resection of the tumor was performed in 13 of 15 patients (87%). Of the patients undergoing resection, major hepatic resection was performed in 8 (62%), while excision of vessels with reconstruction was performed in 5 (38%). Eleven of the 13 resected patients (85%) were discharged from the hospital. Clinical symptoms of recurrent disease occurred between 3 and 36 months after surgery in 7 patients, 6 of whom have died. Three other patients are alive at 5, 21, and 36 months without clinical evidence of recurrence. There was no correlation between the completeness of resection and the duration of disease-free survival.

These results demonstrate that radical resection of high bile duct tumors can be accomplished with an acceptable early mortality rate, thereby extending the benefits of resection to a higher proportion of patients. While resection is clearly effective at controlling local disease and providing palliation of jaundice, surgical cure remains exceptional. Further improvement in the therapy of bile duct cancer must await development of more effective multi-modality approaches.


*HPB Surgery* 1989, Vol. 1, pp. 297–307
Reprints available directly from the publisher
Photocopying permitted by license only

© 1989 Harwood Academic Publishers GmbH
Printed in Great Britain

### EXPERIENCE WITH RADICAL RESECTION IN THE MANAGEMENT OF PROXIMAL BILE DUCT CANCER

JEAN C. EMOND, JAMES T. MAYES, DALE A. ROUCH, J. RICHARD THISTLETHWAITE, and CHRISTOPH E. BROELSCH*

*University of Chicago Pritzker School of Medicine, Department of Surgery, Chicago, IL 60637*

Multiple surgical and nonsurgical approaches have been advocated for the treatment of proximal bile duct cancer. However, survival appears longest when a resection can be performed. Fifteen patients treated at a university center were managed with an aggressive surgical approach. Resection of the tumor was performed in 13 of 15 patients (87%). Of the patients undergoing resection, major hepatic resection was performed in 8 (62%), while excision of vessels with reconstruction was performed in 5 (38%). Eleven of the 13 resected patients (85%) were discharged from the hospital. Clinical symptoms of recurrent disease occurred between 3 and 36 months after surgery in 7 patients, 6 of whom have died. Three other patients are alive at 5, 21, and 36 months without clinical evidence of recurrence. There was no correlation between the completeness of resection and the duration of disease-free survival.

These results demonstrate that radical resection of high bile duct tumors can be accomplished with an acceptable early mortality rate, thereby extending the benefits of resection to a higher proportion of patients. While resection is clearly effective at controlling local disease and providing palliation of jaundice, surgical cure remains exceptional. Further improvement in the therapy of bile duct cancer must await development of more effective multi-modality approaches.

KEY WORDS: Bile duct cancer, hepatic resection, vascular reconstruction.

### INTRODUCTION

Cancers involving the proximal bile ducts have been associated with a poor prognosis. This has resulted in an attitude of "therapeutic nihilism" accepted by many clinicians. Despite the developing consensus in the surgical literature supporting resection of localized lesions[1–6], the convenience of percutaneous or endoscopic biliary drainage has led to widespread use of non-surgical modalitites as primary therapy. While these non-surgical methods have been extremely valuable in the short-term management of biliary obstruction[7–9], long term results have not been critically evaluated. Biliary-enteric anastomosis for the management of malignant biliary obstruction was initially described by Longmire[10] and Dogliotti[11] and later refined by others[12–15]. These experiences with internal biliary drainage have demonstrated improved quality of life during the survival period, avoiding the complications associated with long-term indwelling tube drainage: discomfort, dislodgement, obstruction, and the high frequency of cholangitis.

Although there is universal support for the resection of small, localized lesions, more extensive operations required for the removal of larger tumors have been performed less frequently. The indications for hepatectomy in the removal of bile duct cancers have been recently questioned by Lai *et al.*, citing a mortality rate of 69.2% in their experience[16]. Although several authors have reported favorable experiences with vascular reconstruction in the resection of these lesions[5,17,18], the role of these procedures remains controversial.

In the present report we present our experience with 15 consecutive patients referred for treatment of PBCA. Based on modern concepts of hepatic anatomy and surgery as proposed by Couinaud[19] and extended by Bismuth[20], we attempted to resect the tumor in all cases in which extra-hepatic disease was not present. The overall resection rate was 87%. Vascular reconstruction was required in 5 of 13 resections (38%), while hepatectomy was performed in 8 of 13 (62%). These procedures were accomplished with a hospital mortality rate of 15%. The impact of this aggressive surgical approach on survial and palliation was reviewed.

### PATIENTS AND METHODS:

### *Patient Selection*:

Between January 1985 and June 1988, 15 consecutive patients with proximal bile duct carcinoma were treated. All patients were evaluated at outside hospitals prior to their referral to our institution. Prior biliary drainage had been instituted in 11 of 15 patients, either by percutaneous technique (n = 6) or surgical intubation (n = 5). A single patient had received 6000R of external beam radiation to the liver 8 months prior to evaluation in our institution. Preoperative investigation included standard liver chemistry tests, chest radiographs and abdominal computed tomography in all cases. Diagnostic percutaneous cholangiography was performed unless a recent cholangiogram was available prior to therapy at the outside institution. Arteriography was not performed systematically. Preoperative evidence of systemic malignancy or co-existing medical conditions adding prohibitive surgical risk were regarded as absolute contra-indications to surgery; however, these conditions were absent in all 15 patients. *Surgical Approach*: All patients underwent exploration through a right subcostal incision. Peritoneal carcinomatosis was present in two patients and no further procedures were performed. If localized disease was present, a bilateral subcostal incision was performed. The incision was further extended in the midline to the xiphoid in 10 of 13 patients undergoing resection. In no case was a thoracic incision required. The initial dissection was to encircle the common bile duct below the tumor with skeletonization of the hepatic artery and portal vein at this level. The dissection was then carried superiorly until the extent of vascular involvement and local extension in the hepatic parenchyma could be determined. The intimate relationship between the bile duct and the vessels in the hilus is clearly depicted in figure 1. Local extension of the tumor frequently involved the right hepatic artery, even though local intrahepatic invasion was more common on the left. It was at this point in the dissection that the need for hepatic resection or vascular reconstruction was assessed. Following removal of the specimen, the residual margins of the bile ducts were examined microscopically for completeness of resection. In cases with positive margins, further excision was performed when possible. Vessels were reconstructed using the inferior mesenteric vein, either as a patch in cases of partial excision of the vein wall, or as an interposition graft for arterial reconstruction. Bile duct drainage was accomplished by the construction of Roux-en Y cholangio-jejunostomy in all cases. When intact ducts were present, formal mucosa to mucosa anastomosis was performed, although in four cases high anastomoses were completed using the edge of the hepatic capsule for the anterior wall of the suture line. Trans-hepatic stents were left in 8 cases undergoing high

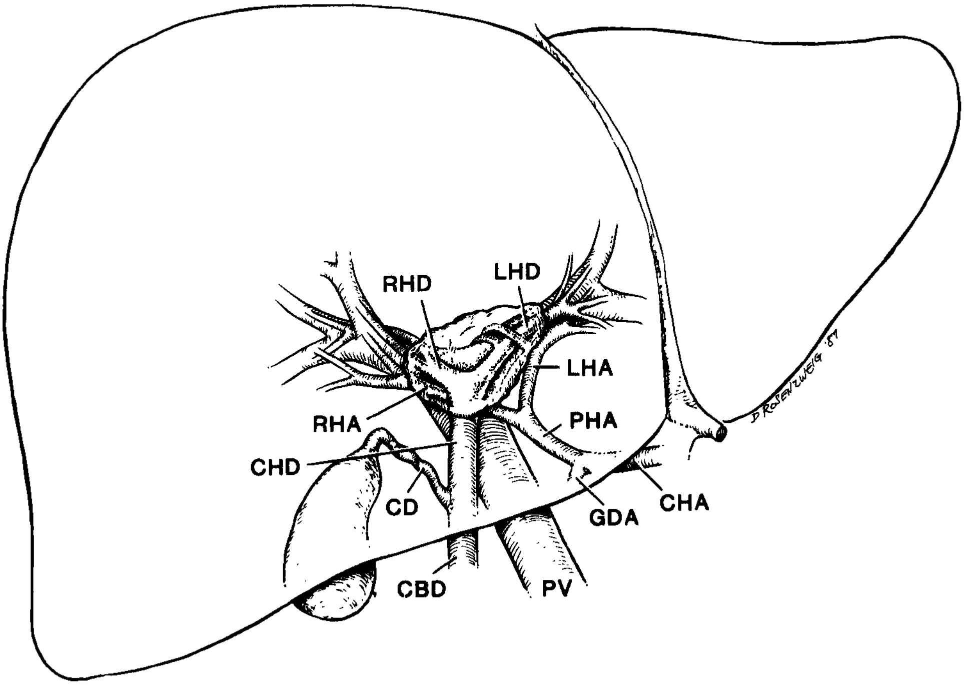

**Figure 1** The anatomic proximity of the bile duct tumor to the hilar vessels is displayed. RHD = right hepatic duct, LHD = left hepatic duct, RHA = right hepatic artery, LHA = left hepatic artery, CHD = common hepatic duct, CD = cystic duct, CBD = common bile duct, PHA = proper hepatic artery, GDA = gastro-duodenal artery, CHA = common hepatic artery, PV = portal vein.

biliary anastomosis, but were removed 6 weeks postoperatively. A single patient with a large tumour underwent total hepatectomy with orthotopic liver transplantation (OLT); a Roux-en-Y choledocho-jejunostomy was performed.

*Postoperative care*: Initially patients were managed in an intensive care setting. Controlled ventilation was maintained until the patients were alert and demonstrated acceptable pulmonary function (usually within 6–24 hours). Patients were able to be transferred to a routine post-surgical ward within 72 hours in all cases.

### RESULTS

### *Descriptive Data*:

Table 1. There were 9 males and 6 females with a median age of 55 years (range 43–67 years). The duration of jaundice ranged from 2 to 16 months and, in 5 patients, chronic abdominal pain preceded the onset of jaundice. One patient had biopsy proven cirrhosis. All patients were in good nutritional condition at the time of assessment. Laboratory examinations revealed good synthetic liver function with a normal prothrombin time in all cases. Serum bilirubin ranged from 0.6 to 18.8 mg/dl. Serum albumin was above 3 g/dl in all but two cases.

*Previous Therapy*:

The impact of previous therapy is demonstrated in Table 2. While surgical intubation was most effective in relieving jaundice, these patients had the highest frequency of cholangitis. Only 1 of 6 patients with prior external biliary drainage had a serum bilirubin less than 5 mg/dl. at the time of evaluation.

*Operative Procedures*:

Table 3 summarizes the procedures in 13 patients undergoing resection. Eight of the 13 patients underwent formal hepatectomy (62%): left hepatectomy was performed in 6 cases, extended right hepatectomy in one, and total hepatectomy with OLT in one. Vascular reconstructions were done in 5 of 13 (38%) patients; one other patient underwent excision of a right hepatic artery without reconstruction. Operative time ranged from 4 to 11 hours and blood loss averaged 5.1 units per procedure (range 1-16 units).

*Early Postoperative Course*:

Nine of the 13 patients (69%) developed a significant complication as listed in Table 4. There was one death 11 days postoperatively from a pulmonary embolus (patient 2). One patient suffered a near fatal pulmonary embulus which required intra-caval filter placement. Four other patients developed clinically significant pulmonary effusions which responded to thoracentesis or tube thoracostomy in one case. Reoperations were performed in 4 cases; 2 after hepatectomy and 2 after bile duct excision without hepatectomy. The patient with previous radiation developed massive intraperitoneal hemorrhage on the seventh postoperative day. At emergency laparotomy the hepatic artery of the remnant right liver was found to be disrupted; the bleeding could not be controlled resulting in intraoperative death. Both patients with carcinomatosis who underwent laparotomy without resection were discharged from the hospital with external biliary drainage catheters in place without complications. Overall, the hospital mortality was 13%; 15% for the 13 who underwent resection. Among the eight patients undergoing hepatectomy mortality was 25%. The median hospital stay was 17.3 days (range 7-30 days).

*Late Follow-up*:

Followup of 13 patients discharged from the hospital has been obtained in all patients as of Sept. 1, 1988. Both patients with carcinomatosis who did not undergo resection died within 3 months after hospital discharge. Late survival for the 11 resected patients is presented in Table 5. Three patients are alive without clinical evidence of disease at 5,21 and 36 months after surgery. Seven patients developed clinical evidence of recurrence between 3 and 36 months postoperatively (median 14 months). Six of these seven patients have died of their disease between 3 and 42 months after surgery (median 18.5 months), while the other is alive with advanced disease at 15 months. Although no quantitative analysis was possible, there was no evident correlation between the duration of survival and the completeness of resection as determined by examination of the surgical specimen. One patient died of a pulmonary embolus 7 months following curative resection. At autopsy, no residual tumor was present. Table 6 presents the clinical findings of the seven patients who

**Table 1** Pre-operative clinical and laboratory findings

| *Observation* | *Value*[a] | |
|---|---|---|
| Number of patients | 15 | |
| Age (yrs) | 55 (43-67) | |
| Duration of symptoms (months) | 4 (2-16) | |
| Sex (M/F) | 9/6 | |
| Previous surgery (present/absent) | 6/9 | |
| Cholangitis (present/absent) | 6/9 | |
| Cirrhosis (present/absent) | 1/14 | |
| Laboratory data: | | |
| AST (I.U./1) | 59 | (10-449) |
| Bili (mg/dl) | 9.4 | (0.6-18.8) |
| Prothrombin time (secs) | 12.5 | (11.4-13.7) |
| Albumin (g/dl) | 3.4 | (2-3.9) |
| Alk phos (I.U./1) | 366 | (127-727) |

a. Categorical data are presented as numbers, continuous data as median, (range).

**Table 2** Effect of previous biliary drainage on the presence of cholangitis and the relief of jaundice.

| *Treatment* | *No. of patients* | *Relief of jaundice*[a] | *Cholangitis* |
|---|---|---|---|
| None[b] | 4 | 0 | 1 |
| Percutaneous drainage | 6 | 1 | 2 |
| Laparotomy with surgical intubation | 5 | 3 | 3 |

a. Serum bilirubin <5 mg./dl
b. One patient underwent laparotomy without biliary drainage.

**Table 3** Operative Procedures in Patients Undergoing Resection

| *Patient* | *Hepatectomy*[a] | *Vascular Repair*[b] | *Biliary repair*[c] | *Curative*[d] |
|---|---|---|---|---|
| 1 | 4,5,6,7,8 | | LHJ | + |
| 2 | 1,2,3,4 | | RHJ | + |
| 4 | 1,2,3,4 | IVC patch | RHJ | |
| 5 | 1,2,3,4 | Portal vein patch | RHJ (2 ducts) | + |
| 6 | Total | Transplant | HJ | + |
| 7 | | RHA (vein graft) | HJ (3 ducts) | + |
| 8 | 1,2,3,4 | | RHJ | + |
| 9 | | | HJ | |
| 10 | 1,2,3,4 | RHA (vein graft) | RHJ (2 ducts) | + |
| 12 | | | HJ | |
| 13 | | RHA ligated | HJ (3 ducts) | |
| 14 | 1,2,3,4 | | RHJ | + |
| 15 | | | HJ (2 ducts) | |

a. Hepatic segments according to Couinaud[21]
b. PV = Portal vein, HA = hepatic artery, IVC = inferior vena cava
c. HJ = hepatico-jejunostomy
d. + = microscopic margins negative

**Table 4** Postoperative course of resected patients

| *Patient* | *Complication* | *Hospital days* | *Survival*[a] |
|---|---|---|---|
| 1. | Pleural effusion | 30 | + |
| 2. | Fatal pulmonary embolus | 11 | |
| 4. | Atelectasis, pleural effusion | 24 | + |
| 5. | | 30 | + |
| 6. | Steroid responsive rejection | 21 | + |
| 7. | | 17 | + |
| 8. | | 8 | + |
| 9. | Reoperation: subphrenic abscess pleural effusion | 30 | + |
| 10. | Reoperation: biliary fistula pleural effusion | 26 | + |
| 12. | Pulmonary embolus | 15 | + |
| 13. | Reoperation: postoperative intestinal obstruction | 21 | + |
| 14. | Reoperation: postoperative hemorrhage | 7 | |
| 15. | | 18 | + |

a. + = discharged home

**Table 5** Late course of patients surviving resection

| *Patient* | *Curative*[a] | *Recurrence*[b] | *Survival*[c] |
|---|---|---|---|
| 1. | yes | 36 | 42 (died) |
| 4. | no | | 36 (alive) |
| 5. | yes | 14 | 19 (died) |
| 6. | yes | 3 | 3 (died) |
| 7. | yes | 16 | 21 (died) |
| 8. | yes | | 21 (alive) |
| 9. | no | 14 | 18 (died) |
| 10. | yes | | 7 (died) |
| 12. | no | 9 | 15 (alive) |
| 13. | no | 3 | 5 (died) |
| 15. | no | | 5 (alive) |

a. Curative = negative microscopic margin of resected specimen
b. Date of clinical recurrence (months).
c. Survival time (months) and status as of Sept. 1, 1988

developed recurrence. Gastro-intestinal complications developed in 4 patients as the first sign of recurrence. These complications occurred between 3 and 16 months postoperatively and were difficult to manage. All four patients subsequently died within 6 months of inanition. Another developed pulmonary metastases. Of interest, none of these five patients developed recurrent jaundice. The patients who received OLT developed diffuse metastatic disease and died 3 months following surgery. Only 1 patient developed jaundice as the first sign of recurrence. This patient has remained symptomatic despite repeated attempts at endoscopic and percutaneous biliary drainage and is currently alive with progressive inanition.

**Table 6** Clinical presentation of late recurrence

| *Patient* | *Time*[a] | *Finding* |
|---|---|---|
| 1. | 36 | Hepatic mass, pulmonary metastases |
| 5. | 14 | Gastric outlet obstruction |
| 6. | 3 | Bone and pulmonary metastases |
| 7. | 16 | Gastric outlet obstruction |
| 9. | 14 | Duodenal hemorrhage |
| 12. | 9 | Cholangitis, jaundice |
| 13. | 3 | Small bowel obstruction, carcinomatosis |

a. Time = months after surgery

### DISCUSSION

The rate of surgical resection in this series is, to our knowledge, the highest reported in the literature on therapy of high bile duct cancers. In addition, the high rate of hepatic resection (62%) and vascular reconstruction (38%) attests to the extensive local involvement of many of the lesions treated. The 15 patients represent a consecutive series, all of whom were referred for treatment from outside hospitals. There was perhaps a bias toward referral of patients with more extensive lesions; however, this selection is typical of all centers with experience in the treatment of biliary tumors. The difficulty of assessing the extent of involvement with preoperative testing also mitigates against the possibility of selective referral. The high resection rate most likely reflects our willingness to perform extensive resection to remove local disease. This was accomplished with a 15% hospital mortality rate for resections, an experience comparable to other published series.

Since the initial description of these tumors as a clinical entity by Klatskin[21], it has been difficult to determine the natural history of the proximal bile duct cancers. In general, slow growth has been characteristic of these lesions, and jaundice usually precedes clinically evident manifestations of systemic disease. This was true in our patients with only 2 of 15 (13%) having peritoneal dissemination at the time of surgery. Since tumor growth is slow, effective treatment of the jaundice is the essential component of palliation. Although short-term control of icterus with external or endoscopic means of biliary drainage is possible[7-9], the quality of life has been poor due to the frequent problems inherent in tube drainage of the biliary tree. In our series (Table 2), preoperative external biliary drainage was rarely successful in clearing jaundice, and although surgical intubation was more effective, the high incidence of cholangitis after surgical intubation is a significant concern. Others, however, have reported more favorable experiences with surgical placement of biliary stents and long-term intubation[6,22,23].

Early postoperative complications were frequent in our patients due to the extensiveness of the operative procedures. Pulmonary effusions were the most frequent but were managed with aspiration in most cases. Pulmonary embolization occurred in the postoperative period in two patients one of whom died. Another patient with a curative resection died 6 months postoperatively from a pulmonary embolus. Although our experience is small, this observation suggests that patients with biliary cancer may be a high-risk group for venous thrombo-embolic complications. The second death in the postoperative period occurred in a patient with previous radiation therapy due to disruption of the hepatic artery. Because of its effects on tissue healing, preoperative radiation may increase surgical risk in patients who require major hepatic resection for removal of the tumor.

Because of the absence of effective systematic therapy of bile duct cancer and the poor results of tube drainage, surgical therapy with internal biliary drainage has become the mainstay of both curative and palliative therapy of these lesions[1-6,12-15]. In our experience resection of bile duct tumors were extremely effective in relieving jaundice. Since excision of involved vessels and liver parenchyma was performed, resection was possible in all the patients with disease confined to the liver, making it possible to establish internal biliary drainage.

While resection for cure is rarely possible, most groups have observed a small number of long term survivors with resection. Significantly improved survival for resected patients is probably one possible using extremely strict criteria which would exclude at least 80% of patients[1]. The decision to perform palliative resection is complicated by the difficulty of assessing the extent of intra-hepatic extension prior to completion of the resection. In favor of palliative resection is the observation of prolonged symptom-free survival in some patients despite the histologic demonstration of residual disease. The impact of our aggressive approach on survival is difficult to determine. Overall follow-up is short in the present series; however, no patient appears to have been cured. As demonstrated in Table 5, most patients with palliative resection were disease-free for at least one year. The earliest recurrence occurred in the patient receiving OLT. Recurrence was most often associated with gastrointestinal symptoms (Table 6), either due to duodenal obstruction or malignant intestinal obstruction associated with carcinomatosis. These patients were difficult to manage and usually survived several months with chronic pain and development of progressive inanition.

In conclusion, the primary advantage of our approach of radical surgical excision is the extension of the possibility of cure or prolonged palliation to a larger proportion of patients with bile duct cancer. All resected patients were free of jaundice at the time of discharge and enjoyed symptom free survival for varying intervals. Although our experience with liver transplantation for biliary cancer is confined to a single patient, the experience of early systemic spread despite an apparently curative resection is consistent with other observations of OLT in malignant disease[24,25]. At present, based on our experience and a review of the literature, resection of the lesion with conventional surgical techniques for removal of gross disease provides effective palliation and, additionally, the chance is cure in a small proportion of patients. Postoperative radiation therapy, either using external beam or intra-biliary stents[26], may have added benefit although the impact of these modalities is difficult to determine due to variability of the natural history of this disease. More effective therapy of bile duct cancer must clearly await progress in radiation or chemotherapeutic modalities to supplement surgical removal.

### *References*

1. Bismuth, H., Castaing, D. and Traynor, O. (1988). Resection or palliation: priority of surgery in the treatment of hilar cancer. *World Journal of Surgery*, **12**, 39–47.

2. Evander, A., Fredlund, P., Hoevels, J., Ihse, I. and Bengmark, S. (1980) Evaluation of agressive surgery for carcinoma of the extrahepatic bile ducts. *Annals of Surgery*, **191**, 191-223.
3. Launois, B., Campion, J.P., Brissot, P. and Gosselin. (1979) Carcinoma of the hepatic hilus. Surgical management and the case for resection. *Annals of Surgery*, **190**, 151-157.
4. Fortner, J.G., Kallum, B.O. and Kim, D.K. (1976) Surgical management of carcinoma of the junction of the main hepatic ducts. *Annals of Surgery*, **184**, 68-73.
5. Blumgart, L.H., Benjamin, I.S., Hadjis, N.S. and Beazley, R. (1984) Surgical approaches to cholangiocarcinoma at the confluence of hepatic ducts. *Lancet*, **i**, 66-70.
6. Cameron, J.L., Broc, P. and Zuidema, G.D. (1982) Proximal bile duct tumors. *Annals of Surgery*, **196**, (4) 412-419.
7. Cotton, P.B. (1982) Duodenoscopic placement of biliary prostheses to relieve malignant obstructive jaundice. *British Journal of Surgery*, **69**, 501-503.
8. Huibregtse, K. and Tytgat, G.N. (1982). Palliative treatment of obstructive jaundice by transpapillary introduction of large bore bile duct endoprosthesis. *Gut*, **23**, 371-375.
9. Freeny, P.C., Gannon, R.M. and Ball, T.J. (1982) Endoscopic retrograde biliary drainage. *American Journal of Radiology*, **139**, (8) 399-401.
10. Longmire, W.P. and Sanford, M.C. (1948) Intrahepatic cholangiojejunostomy with partial hepatectomy for biliary obstruction. *Surgery*, **24**, 264-76.
11. Dogliotti, A.M. (1951) Zur operations technik bei verschluss der extrahepatischen gallenwege. Langenbecks. *Arch. Klin. Chir.*, **270**, Kongrassbericht, 101-103.
12. Soupault, R. and Caroli J. (1955) Cancer du canal hepatique. Un nouveau procede d'hepato-cholangio-jejunostomie. *Memoirs de l'Academie de Chirurgie*, **81**, 902-908.
13. Bismuth, H. and Corlette M.B. (1975) Intrahepatic cholangioenteric anastomosis in carcinoma of the hilus of the liver. *Surgery, Gynecology and Obstetrics*, **140**, (1) 170-178.
14. Cameron, J.L., Gaylek, B.W. and Harrington, D.P. (1978) Modification of the Longmire procedure. *Annals of Surgery*, **187**, (4) 379-382.
15. Malt, R.A., Warshaw, A.L., Jamieson, C.H. and Hawk J.C. (1980) Left intrahepatic cholangiojejunostomy for proximal obstruction of the biliary tract. *Surgery, Gynecology and Obstetrics*, **150**, (2) 193-197.
16. Lai, E.C.S., Tompkins, R.K. Roslyn, J.J. and Mann L.L. (1987) Proximal bile duct cancer. *Annals of Surgery*, **205**, (2) 111-118.
17. Sagakuchi, S. and Nakamura, S. (1986) Surgery of the portal vein in resection of cancer of the hepatic hilus. *Surgery*, **99**: 344-349.
18. Lygidakis, N.J., van der Heyde, M.N., van Dongen, R.J., Kromhout, J.G., Tytgat, G.N. and Huibregtse, K. (1988) Surgical approaches for unresectable primary carcinoma of the hepatic hilus. *Surgery, Gynecology and Obstetrics*, **166**: 107-114.
19. Couinaud, C. (1957) Le foie: etudes anatomiques et chirurgicales. Paris: Masson.
20. Bismuth, H. (1982) Surgical anatomy and anatomical surgery of the liver. *World Journal of Surgery*, **6**: 3-9.
21. Klatskin, G. (1965) Adenocarcinoma of the hepatic duct at its bifurcation with the porta hepatis. *American Journal of Medicine*, **38**, (2) 241-256.
22. Praderi, R.C., Estefan, A.F. and Tiscornia, E. (1985) Transhepatic intubation in benign and malignant lesions of the biliary ducts. Current Problems in Surgery, Ravitch M.M, ed., **22**: (12).
23. Terblanche, J., Saunders, S.J. and Louw, J.H. (1972) Prolonged palliation in carcinoma of the main hepatic duct junction. *Surgery*, **71**, (5) 720-731.
24. Calne, R.Y. (1982) Liver transplantation for liver cancer. *World Journal of Surgery*, **6** (1) 76-80.
25. Iwatsuki, S.L., Klintmalm, G.B. and Starzl, T.E. (1982) Total hepatectomy and liver replacement (orthotopic liver transplantation) for primary hepatic malignancy. *World Journal of Surgery*, **6**, 81-85.
26. Conroy, R.M., Shahbazian, A.A., Edwards, K.C., Moran, E.M., Swingle, K.F., Lewis G.J. and Pribram F.W. (1982) A new method for treating carcinomatous biliary obstruction with intracatheter radium. *Cancer*, **49**, (7) 1321-1327.

*Accepted by S. Bengmark on 6 November 1988*

### INVITED COMMENTARY

The authors recommend radical surgical resection of proximal bile duct cancer whenever possible provided that it can be accomplished with a low operative mortality rate because they believe this offers the best survival potential and quality of life. In 10 patients, 9 of whom underwent resection, with a mean follow-up of 12.5 months, they report a 50% disease free survival and a 70% survival rate. The quality of survival is difficult to discern from the data presented.

The Cape Town group have recently presented a different viewpoint based on long-term follow-up of a consecutive group of patients treated palliatively, usually with U-tube intubation[1]. This summary of the Cape Town data and conclusions is presented to help achieve perspective in assessing controversies surrounding the management of high bile duct carcinoma. Our group studied 21 patients with cholangiocarcinoma at the confluence of the main right and left hepatic ducts seen consecutively in the period from 1968 to 1982 and evaluated, treated and documented prospectively with follow-up until mid-1986. All tumours were deemed irresectable. The U-tube palliative bypass was developed during this study and used in 14 patients. Histological confirmation of the diagnosis was obtained in 71% patients and seven patients received radical radiotherapy as additional treatment. The 30 day overall hospital mortality was 19% with a one and two year actual survival of 57% and 33% respectively. The quality of life was usually good. The Cape Town paper concluded that radical resection was seldom possible and advocated U-tube palliative management in the majority of patients[1].

The need to obtain histological confirmation of the diagnosis is generally accepted today. The rare papillary tumours, which tend to grow into the lumen of the bile duct and which have a better prognosis, must be resected whenever possible. The question is whether radical resection is able to eradicate the usual scirrhous type carcinoma. The answer is probably 'no' in most instances thus the role of radical resection must be questioned, unless it can be accomplished with minimal mortality as in the Chicago study. Weinbren and Mutum studied resected material at the Hammersmith Hospital, London, and emphasised the tendency of this carcinoma to spread locally, often beyond the confines of the macroscopic limits of the tumour. Nerve involvement was common and the growth tended to spread to the liver between the hepatocyte plates and also along the bile ducts both within the wall and at times beneath apparently normal intact lining epithelial cells. The biological nature of this carcinoma which tends to spread beyond the confines of radical local surgery[2], may well ultimately defeat the surgeon's enthusiasm for resection. In reviewing a recent paper Adson summarised the situation by stating that in the setting of a condition that is seldom cured by heroic resection one has to "decide whether what can be done, should or should not be done"[3].

The management options remain controversial in 1988. There have been a surprising number of reports advocating radical resection for this tumour[1]. The alternatives to radical resection are operative dilatation with intubation either using a U-tube as advocated by the Cape Town group[1], or a straight tube as recommended by others[4], or operative bypass using the round ligament approach or finally endoprosthesis insertion. Any of these techniques may be combined with radical radiotherapy. The author favours radiotherapy although the evidence remains circumstantial[1]. Endoprostheses have proved problematic because of the long survival of these patients leadcing to repeated blockage together with the difficulty of insertion in high bile duct lesions. Liver transplantation remains unjustified because of disappointing results due to recurrence of tumours when compared with the excellent palliation that can be achieved with simple therapy. In assessing the results of radical resection it must be remembered that almost all of the enthusiastic reports have had a short follow-up and that with longer follow-up an increasing number of the patients ultimately die of the underlying tumour[6]. The longterm follow-up from Los Angeles has indicated that the quality of survival is similar with either curative resection or intubation[7]. Two major American studies have emphasised that resection of the bile duct is only justified if this can be achieved without hepatic resection[4,7].

Our group have reported the results of palliative therapy in consecutive unselected patients including operative dilatation and U-tube placement with or without radiotherapy and demonstrated as good survival as that achieved in many major series of selected patients undergoing radical resection[1]. The author concedes that the only chance of cure is radical surgical resection but believes that this is seldom possible. He agrees with the Los Angeles group that resection should only be recommended when it can be performed without concomitant hepatic resection[7]. Both easily resectable lesions and patients with papillary carcinoma are unfortunately relatively uncommon but must be sought out. If good results are to be achieved, this difficult group of patients must be referred to specialist centres with a particular interest in high bile duct carcinoma.

### *References*

1. Terblanche J., Kahn, D., Bornman P.C., Werner D. (1988). The role of the U-tube palliative treatment in high bile duct carcinoma. *Surgery*, in press.
2. Weinbren K., Mutum, S.S. (1983). Pathological aspects of cholangiocarcinoma. J. Path. **139**, 217-38.
3. Beazley, R.M., Hadjis, N., Benjamin, I.S., Blumgart, L.H. (1984). Clinicopathological aspects of high bile duct cancer. Experience with resection and bypass surgical treatments. *Ann. Surg*. **199**, 623-35.
4. Cameron, J.L., Broe, P., Zuidema, G.D. (1982). Proximal bile duct tumours. Surgical management with silastic transhepatic biliary stents. *Ann. Surg*. **196**, 412-9
5. Bismuth, H., Corlette, M.B. (1975). Intrahepatic cholangioenteric anastomosis in carcinoma of the hilus of the liver. *Surg. Gynec. Obstet*. **140**, 170-8.
6. Hadjis, N.S., Benjamin, I.S., Blumgart, L.H. (1986). Results of the agressive surgical approach to hilar cholangiocarcinoma. *Gut*. **27**, 1279 (Abstract).
7. Lai, E.C.S., Tomkins, R.K., Roslyn, J.J., Mann, L.L. (1987). Proximal bile duct cancer. Quality of survival. *Ann. Surg*. **205**, 111-8.

John Terblanche
University of Cape Town
Medical School OBS 7925
Cape Town
South Africa

